# A novel esterase from a soil metagenomic library displaying a broad substrate range

**DOI:** 10.1186/s13568-021-01198-5

**Published:** 2021-03-05

**Authors:** Jian Yao, Lun Gui, Shaocheng Yin

**Affiliations:** Institute of Agricultural Applied Microbiology, Jiangxi Academy of Agricultural Sciences, Nanchang, 330200 People’s Republic of China

**Keywords:** Multi‐functional esterase, Tannase, Chlorogenate esterase, Feruloyl esterase

## Abstract

A novel esterase gene was isolated from a soil metagenomic library. The gene encoded a protein of 520 amino acids which contained a 21 aa signal peptide. Primary structure analysis of the protein sequence revealed that it contained a conserved active site motif (SxSxG) and a structural motif (CS-D-HC). Then the esterase gene was cloned and expressed in *Escherichia c*oli BL21(DE3). SDS-PAGE analysis of the purified esterase showed that it was expressed in a highly soluble form and its molecular mass was estimated to be 55 kDa. Characterization of the esterase revealed that it exhibited high activity toward *p*-nitrophenyl esters with short acyl chains and especially *p*-nitrophenyl acetate, suggesting that it was a typical carboxylesterase rather than a lipase. With *p*-nitrophenyl acetate as substrate, the enzyme showed its optimal activity at pH 7.0 and 30 °C, and it was stable at a broad pH range from 4.5 to 10.0 and temperature not higher than 50 °C. Furthermore, the enzyme showed different substrate specificity from known esterase, it was not only hydrolyzing against *p*-nitrophenyl esters, but also hydrolyzing all hydroxybenzoic esters and hydroxycinnamic ester assayed. As it was an enzyme active on a broad range of phenolic esters, simultaneously possessing feruloyl esterase, chlorogenate esterase and tannase activities, it could serve as a valuable candidate for applications in biotechnology.

**Supplementary Information:**

The online version contains supplementary material available at 10.1186/s13568-021-01198-5.

## Introduction

Plant cell wall polysaccharides, the major reservoir of fixed carbon in nature, had been divided into three main groups: cellulose, hemicellulose and lignin (Kosugi et al. 2001). In addition to these main groups, phenolic compounds were thought to be playing a key role in structure of the plant cell wall as they covalently cross-linked plant cell wall polysaccharides to each other by ester bonds (Ishii 1997; Nieter et al. 2015). Furthermore, phenolic compounds were also reported to protect plants from oxidative stress, UV radiation, invasion and infection (Jaleel et al. 2009). Thus, these phenolic compounds not only influenced the rigidity and mechanical properties of the cell wall but also played a role in plant defense (Nieter et al. 2015; Cosgrove 2001). Phenolic compounds showed a large diversity of structures, from simple molecules e.g. phenolic acids to polymeric compounds based on these different classes (Cheynier 2012). Hydroxycinnamic acids (such as caffeic, ferulic, *p*-coumaric and chlorogenic acids) and hydroxybenzoic acids (such as tannins), two classes of typical phenolic acids, were derived from benzoic and cinnamic acids, respectively (Koseki et al. 2010). With the in-depth study, the industrial use of phenolic acids had attracted growing interest because of their potential biological properties, such as antioxidant, chelating, free radical scavenging and anti-inflammatory (Rice-Evans et al. 1996; Natella et al. 1999; Silva et al. 2000; Castelluccio et al. 1996). Therefore, the removal of these phenolic compounds and the breakdown of ester linkages between polymers allowed numerous exploitations for pharmaceuticals industrial and food applications.

Lipolytic enzymes, including esterases and lipases, belonged to the general class of carboxylic ester hydrolases (EC 3.1.1) that catalyzed both the hydrolysis and formation of ester bonds. While esterases (carboxylic acid esterases, EC 3.1.1.1) hydrolyzed water-soluble or emulsified esters with short chain carboxylic acids with less than 10 carbons, lipases (EC 3.1.1.3) preferred long-chain fatty acids with more than 10 carbons (Gao et al. 2016). Feruloyl esterases (FAEs, E.C. 3.1.1.73), represented a subclass of the carboxylic acid esterases, could enable and facilitate the access of hydrolases to the backbone wall polymers. With synergy of xylanases, cellulases and pectinases, feruloyl esterases had been used as a key enzyme to break down complex plant cell wall carbohydrates (Crepin et al. 2003; Faulds and Williamson 1995; Kroon and Williamson 1996). Other carboxylic acid esterases, tannase (E.C. 3.1.1.20) catalyzed the hydrolysis of ester and depsidic linkage in hydrolysable tannins releasing gallic acid and glucose, while chlorogenate esterase (E.C. 3.1.1.42) catalyzed the hydrolysis of the ester bond presented in chlorogenic acid (Ramírez et al. 2008). Up to date, these types of carboxyl ester hydrolases from different organisms had been isolated, characterized and some of them were industrially produced. However, the use of carboxyl ester hydrolases with new specificities was still needed (Tutuncu et al. 2019). In the view of high demand for these biocatalysts in biotechnology, new experimental approaches had been developed over last few years, such as metagenomic which was a cultivation-independent method recovering directly total DNA of culturable and uncultured microorganisms from environmental samples for discovering novel enzymes with unique bioactivities.

In this study, a novel esterase (Tan410) gene was isolated from a soil metagenomic library by function-driven screening approach. The esterase gene was cloned, expressed in *E.coli* BL21(DE3) and purified for investigating biochemical characteristics of the recombinant enzyme. From the esterase assayed, Tan410 was not only able to hydrolyze phenolic ester, but also able to hydrolyze hydroxycinnamic and hydroxybenzoic ester, indicating that a novel esterase with a novel function was isolated from a soil metagenomic library.

## Materials and methods

All chemicals (phenyl acetate, ethyl acetate, propyl acetate, butyl acetate, isobutyl acetate, ethyl butyrate, ethyl hexanoate, ethyl octanoate, ethyl decanoate, ethyl oleate, vinyl acetate, vinyl butyrate, vinyl decanoate, vinyl pivalate, isopropenyl acetate, glyceryl triacetate, glyceryl tributyrate, methyl bromoacetate, ethyl bromoacetate, methyl glycolate, ethyl gallate, propyl gallate, methyl benzoate, ethyl benzoate, vinyl benzoate, methyl 4-hydroxybenzoate, ethyl 4-hydroxybenzoate, methyl vanillate, methyl 2,4-dihydroxybenzoate, methyl 2,5-dihydroxybenzoate, methyl 3,4-dihydroxybenzoate, methyl 3,5-dihydroxybenzoate, methyl ferulate, methyl caffeate, methyl *p*-coumarate, methyl sinapinate, chlorogenic acid, caffeic acid, ferulic acid, gallic acid, cinnamic acid, vanillic acid, benzoic acid, sinapic acid, 4-hydroxybenzoic, 2,4-dihydroxybenzoic acid, 3,4-dihydroxybenzoic acid, 2,5-dihydroxybenzoic acid and 3,5-dihydroxybenzoic acid) were purchased from Aladdin (Shanghai China) and Sigma-Aldrich (Taufkirchen, Germany) unless otherwise noted.

### Metagenomic library construction, esterase gene screening and sequence analysis

Metagenomic DNA extraction from soil and metagenomic library construction was based on the method described previously (Yao et al. 2011). 5-bromo-4-chloro-3-indolyl-β-d-indolyl caprylate was used as selection substrate for esterase gene screening. Clones with blue transparent circles around was chosen for reconfirmed and sub-cloned. DNA sequencing was carried out by using an ABI Prism 377 DNA sequencer (Applied Biosystems, Inc.) and sequence analysis was performed by online server Open Reading Frame (ORF) Finder (.nih.gov/orffinder/) (Hancock and Bishop 2004). Amino acid sequence alignment of homologous esterases was carried out by Clustal W program (Larkin et al. 2007). Protein functional domains of esterase were analyzed by comparing its sequence with those in the NCBI and Pfam databases. Molecular weight and p*I* of the protein was predicted by ProtParam tool as implemented in ExPASy (Gasteiger et al. 2005).

### Expression and purification of recombinant esterase

Gene encoding Tan410 was amplified using primers f 1 (5′-CGCGGATCCATGCCCGCAAAAACCCGCCTG-3′) and w1 (5′-CCCAAGCTTATTCCCGTTAGTAAAGCCGTC-3′) (restriction sites underlined for *Bam*HI and *Hin*dIII, respectively). PCR products were purified and cloned into restriction digested pET-28a (+) and transformed into *E.coli* BL21(DE3). Positive clone was grown in a 500-mL flask containing 300 mL LB at 37 °C until the cell concentration reached an OD_600_ of 0.8, and then were induced with 0.4 mM IPTG at 25 °C (12 h, 180 rpm). Culture cells were harvested by centrifugation (12,000×*g*, 5 min) and resuspended with 30 mL lysis buffer (50 mM NaH_2_PO_4_, 300 mM NaCl, 5 mM imidazole, pH 8.0). Then cells were sonicated and supernatant was collected by centrifugation at 4 °C (16,000×*g*, 15 min). Subsequently, the supernatant incubated with 5 mL Ni–NTA resin in a closed column for 1 h on a rocking shaker at 4 °C. Purification was performed according to the manufacturer’s instructions. The column was washed two times with 30 mL wash buffer I (50 mM NaH_2_PO_4_, 300 mM NaCl, 10 mM imidazole, pH 8.0), followed by 30 mL wash buffer II (50 mM NaH_2_PO_4_, 300 mM NaCl, 20 mM imidazole, pH 8.0). Target protein was eluted with 5 mL elution buffer (50 mM NaH_2_PO_4_, 300 mM NaCl, 250 mM imidazole, pH 8.0), and elution fractions were rebuffered with 50 mM phosphate buffer (pH 7.0). The purification was monitored using denaturing SDS-PAGE analysis (12% separating gel and 5% concentrating gel) according to Laemmli (Laemmli 1970).

### Spectrophotometric assays of substrate specificity and esterase activity

Substrate specificity of purified Tan410 was determined in 50 mM phosphate buffer (pH 7.0) containing 1 mM of *p*-nitrophenyl esters such as *p*-nitrophenyl acetate (C2), *p*-nitrophenyl butyrate (C4), *p*-nitrophenyl caproate(C6), *p*-nitrophenyl caprylate (C8), *p*-nitrophenyl decanoate (C10) under standard conditions (30 °C for 5 min). Stock substrate solutions (30 mM) were prepared by dissolving *p*NP-esters in pure methanol.

Esterase activity was performed with *p*NP-esters as substrates. Reactions containing 0.1 mL of enzyme solution and 0.1 mL of 5 mM substrate in 50 mM Tris-HCl (pH 7.0) were incubated at 30 °C for 5 min. Production of *p*-nitrophenol was measured at 410 nm. One unit (U) of esterase activity was defined as the amount of enzyme that released 1 µmol of *p*-nitrophenol per minute.

Substrate profile of purified Tan410 was determined by using an ester library (phenyl acetate, ethyl acetate, propyl acetate, butyl acetate, isobutyl acetate, ethyl butyrate, ethyl hexanoate, ethyl octanoate, ethyl decanoate, ethyl oleate, vinyl acetate, vinyl butyrate, vinyl decanoate, vinyl pivalate, isopropenyl acetate, glyceryl triacetate, glyceryl tributyrate, methyl bromoacetate, ethyl bromoacetate and methyl glycolate) with *p*-nitrophenol used as a pH indicator to monitor ester hydrolysis colorimetrically (Esteban-Torres et al. 2013). Blank reactions with 1 mM sodium phosphate buffer (pH 7.0) were carried out for each substrate.

Esterase activity on gallate esters (tannase activity) was determined using a rhodamine assay specific for gallic acid (Inoue and Hagerman 1988). And the amount of gallic acid in reaction mixture was determined as described previously (Yao et al. 2011). One unit of tannase activity was defined as the amount of enzyme required to release 1 µmol of gallic acid per minute.

### Effect of temperature and pH on esterase activity

The optimum temperature for esterase activity of Tan410 was assayed by incubating the enzyme in 50 mM phosphate buffer (pH 7.0) at different temperatures in a range of 25–65 °C. Thermostability of Tan410 was investigated by pre-incubating the enzyme at 25–65 °C for 1 h and then analyzing the residual activity. The optimal pH values of Tan410 was measuring its activity at different pH values (4.0–9.0). Following buffers were used for the assay: 50 mM sodium acetate buffer (pH 4.0–6.0); 50 mM phosphate buffer (pH 5.5–8.0); 50 mM Tris-HCl buffer (pH 7.5–9.0). To study pH stability, Tan410 was incubated in different buffers (pH4.0–12.0) for 1 h. Then the pre-incubated solution was regulated to pH 7.0 with 500 mM phosphate buffer and analyzed for the residual activity.

### Effect of metal ions, detergents, inhibitor and organic solvents on esterase activity

Effects of metal ions (Mg^2+^, Mn^2+^, Cu^2+^, Co^2+^, Zn^2+^, K^+^, Fe^2+^, Ni^2+^, Ca^2+^, Ag^+^ and Pb^2+^), detergents [CTAB (Cetyltrimethylammonium bromide) and SDS (Sodium dodecyl sulfate)] and inhibitor phenylmethylsulfonyl fluoride (PMSF) on esterase activity were investigated. Reaction solutions were assayed by incubating Tan410 and various metal ions (5 mM) for 1 h (at 4 °C) in 50 mM phosphate buffer (pH 7.0) and then analyzing the residual activity. To estimate the organic solvent tolerance of Tan410, enzyme solutions were mixed with various organic solvents (*N*,*N*- dimethyl formamide, ethyl acetate, isopropyl alcohol, isobutyl alcohol, isoamyl alcohol, acetone, methanol and ethanol) at a final concentration of 10%, and incubated at 4 °C for 1 h. Then the residual activity was assayed. Esterase activity measured in absence of any additive was taken as a control and was given a value of 100%. The experiments were done in triplicate.

### HPLC analysis of Tan410 activity on phenolic esters

Activity of Tan410 against 17 potential substrates was analyzed by HPLC (high-performance liquid chromatography). Substrates assayed were esters derived from benzoic and hydroxycinnamic acids. Among benzoic acids, benzoic esters (methyl benzoate, ethyl benzoate and vinyl benzoate), hydroxybenzoic esters (methyl 4-hydroxybenzoate and ethyl 4-hydroxybenzoate), vanillic ester (methyl vanillate), dihydroxybenzoic esters (methyl 2,4-dihydroxybenzoate, methyl 2,5-dihydroxybenzoate, methyl 3,4-dihydroxybenzoate and methyl 3,5-dihydroxybenzoate) and gallic esters (ethyl gallate and propyl gallate) were analyzed. In relation to hydroxycinnamic acids, ferulic ester (methyl ferulate), caffeic ester (methyl caffeate), *p*-coumaric ester (methyl *p*-coumarate), sinapic ester (methyl sinapinate) and chlorogenic acid were analyzed. Enzyme solutions mixed with substrates (1 mM) were incubated at 30°C for 30 min. As controls, phosphate buffer containing reagents but not the enzyme was incubated under the same condition. Reactions were terminated by adding acetonitrile and formic acid and then analyzed by HPLC-UV method. Samples of reaction were centrifuged (16,000*g*×20 min) before injection into HPLC column. Aliquots (5 µL) of the reaction mixture were loaded onto the HPLC system (Agilent 1260 Infinity II, USA) using a 150 × 3.0 mm Proshell 120 column (Agilent, USA). Substrates and products were separated by using a stepwise gradient at a flow velocity of 1.0 mL min^− 1^ as follow: 10 to 30% acetonitrile in 10 min. 30 to 56% acetonitrile in 1.5 min, 56 to 96% acetonitrile in 0.5 min, 96 to 99% acetonitrile in 2.5 min, 99 to 15% acetonitrile in 0.5 min, 15 to 10% acetonitrile in 1 min, and re-equilibration with 10% acetonitrile for 1 min, for a total run time of 17 min. Substance elution was detected at 232 nm (benzoic, 2,4-dihydroxybenzoic acid, 2,5-dihydroxybenzoic acid, 3,4-dihydroxybenzoic acid and 3,5-dihydroxybenzoic acid), 254 nm (4-hydroxybenzoic and vanillic acids), 275 nm (cinnamic and gallic acids), 306 nm (*p*-coumaric acid), and 323 nm (ferulic, caffeic, chlorogenic and sinapic acids).

### Nucleotide sequence accession number

The nucleotide sequence reported in this study had been submitted to GenBank under the accession number HQ147564.

## Results

### Esterase screening and sequence analysis

A metagenomic library containing 9.2 × 10^4^ clones with insert DNA sizes ranging from 2.5 kb to 5.0 kb was constructed for discovering esterase genes. Functional screening of the metagenomic library based on hydrolysis of 5-bromo-4-chloro-3-indolyl-β-d-indolyl caprylate resulted in the detection of a positive clone, forming a clear zone (bule color) on indicator plate. Sequence analysis revealed that the insert DNA fragment was 5335 bp and harbored 21 possible functional ORFs as revealed by ORF Finder program, in which one ORF consisting of 1563 bp was identified as a putative esterase named Tan410. Molecular weight and theoretical p*I* of the enzyme was predicated to be 54.88 kDa and 4.66, respectively. Analysis of the sequence using SignalP 5.0 predicted the first 21 amino acids of the protein served as a signal peptide (Sec/SPI), suggesting that Tan410 was naturally a secreted enzyme. It was also found that Tan410 belonged to Pfam07519 family of the alpha/beta-hydrolases with a catalytic triad (serine, glutamate or aspartate, and histidine). Alignment of Tan410 with homologous sequences revealed that an active site motif Ser-X-Ser-X-Gly and a structural motif CS-D-HC in Tan410 sequence. (see Additional file 1: Figure S1).

### Expression and purification of recombinant esterase Tan410

Gene *tan410* was ligated to vector pET-28a (+) and expressed in *E .coli* BL21 (DE3) for biochemical properties characterization. SDS-PAGE analysis of the purified enzyme showed a single band corresponding to about 55 kDa which was in accordance with the theoretical molecular mass (see Additional file 2: Figure S2).

### Biochemical properties of esterase Tan410

**Fig. 1:**
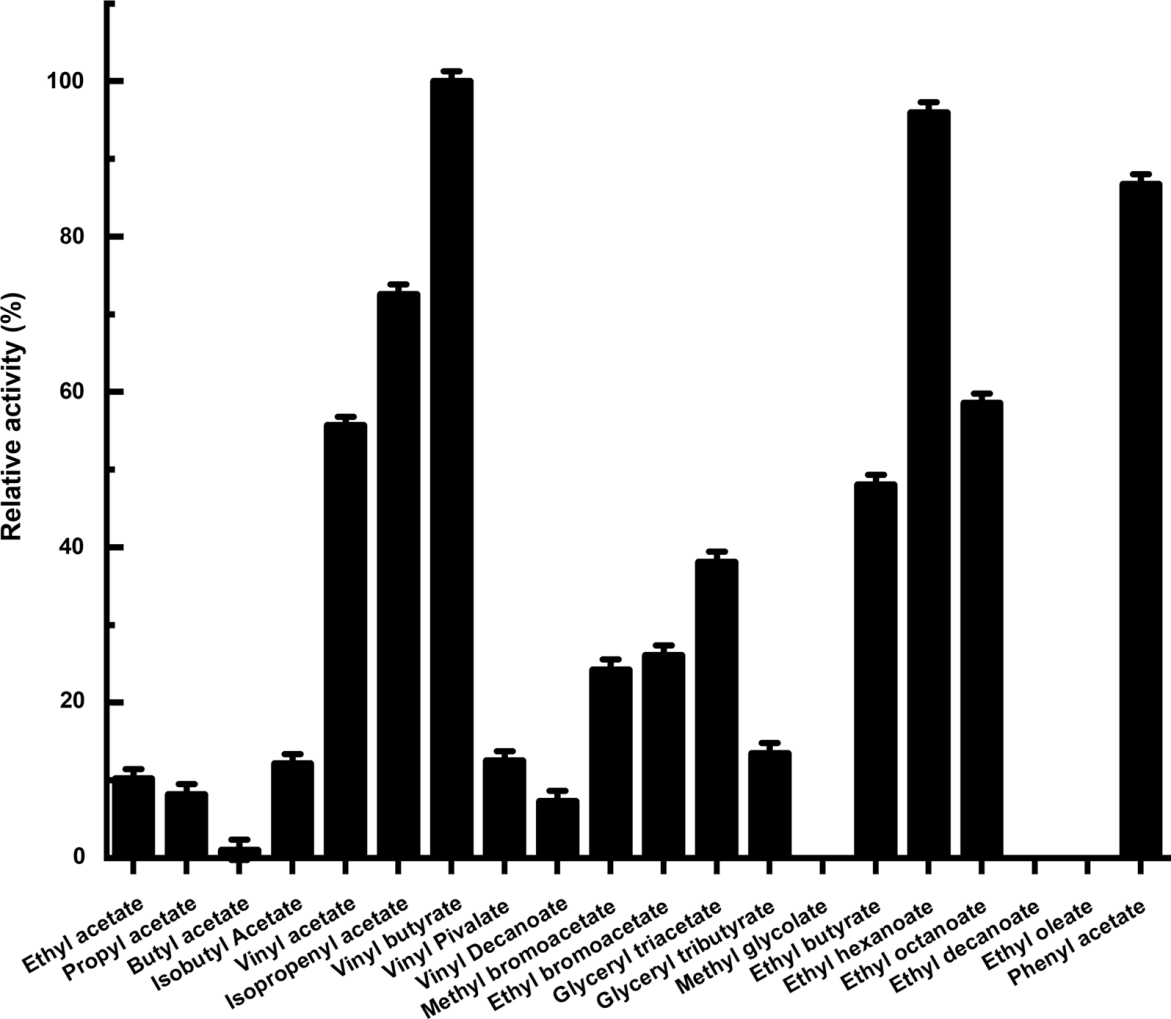
Substrate profile of esterase Tan410

**Fig. 2:**
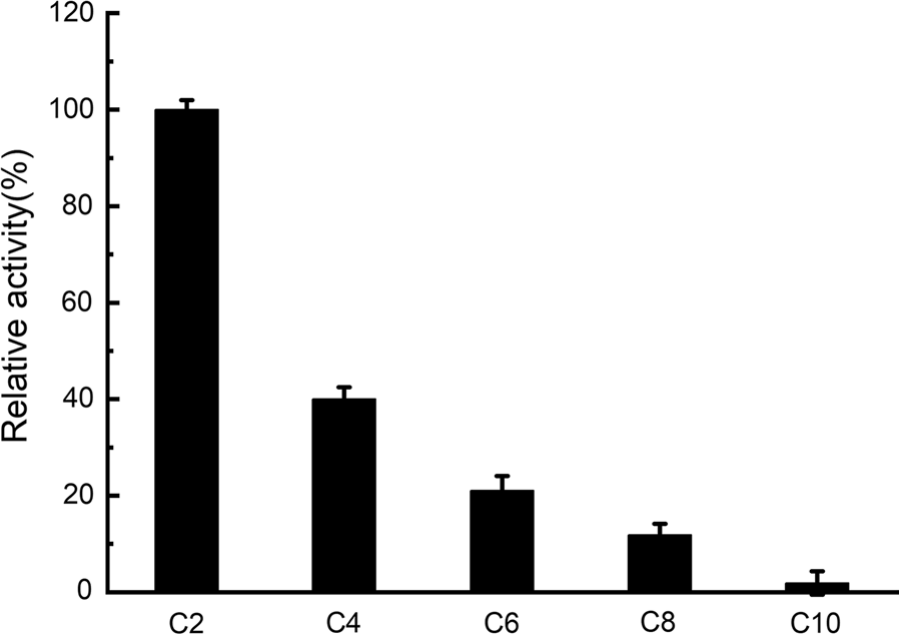
Substrate specificity of esterase Tan410 toward *p*-nitrophenyl esters

**Fig. 3:**
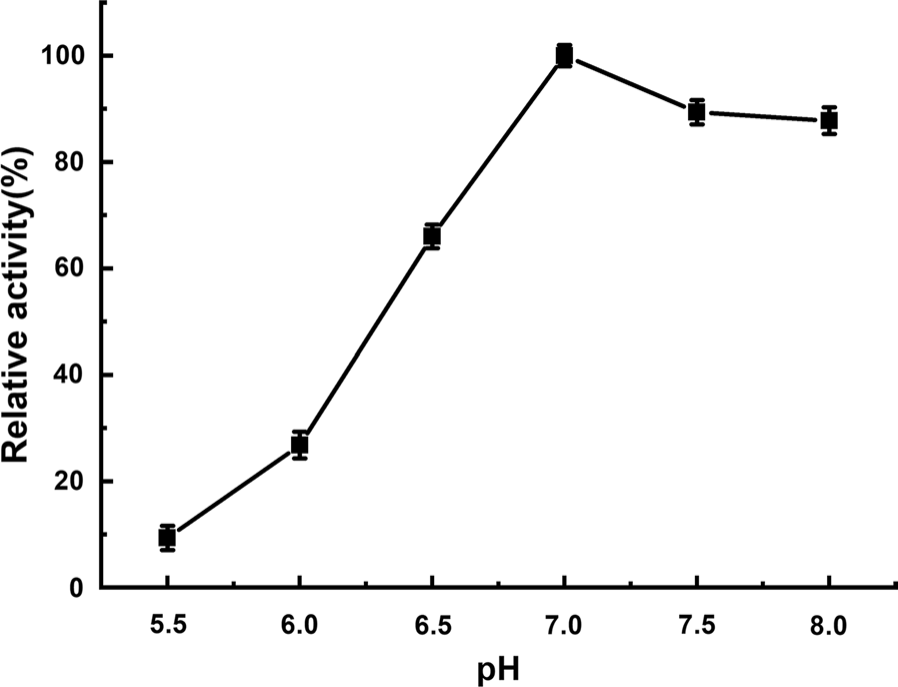
Effect of pH on activity of esterase Tan410

**Fig. 4:**
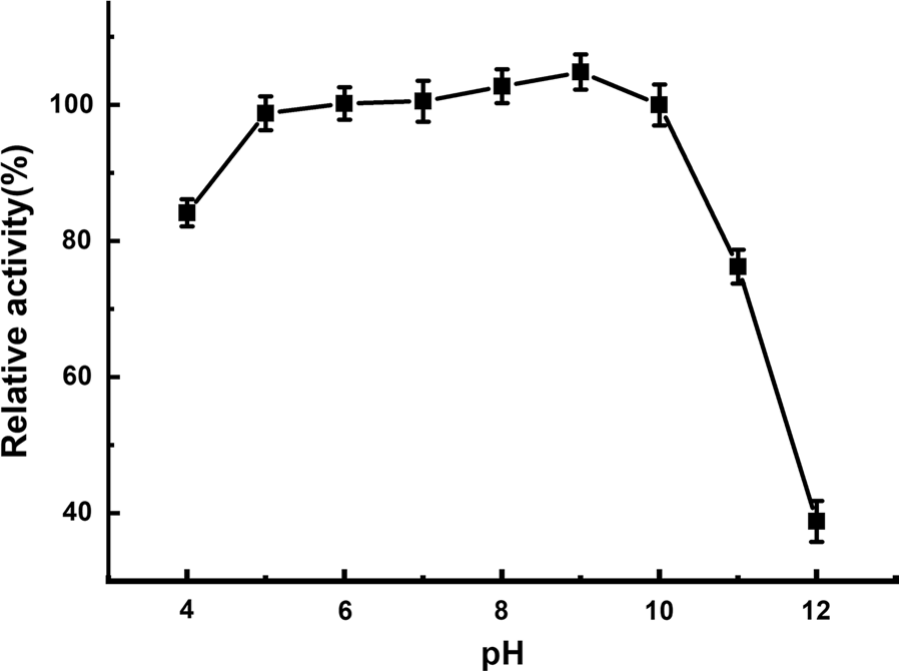
Effect of pH on the stability of esterase Tan410

**Fig. 5:**
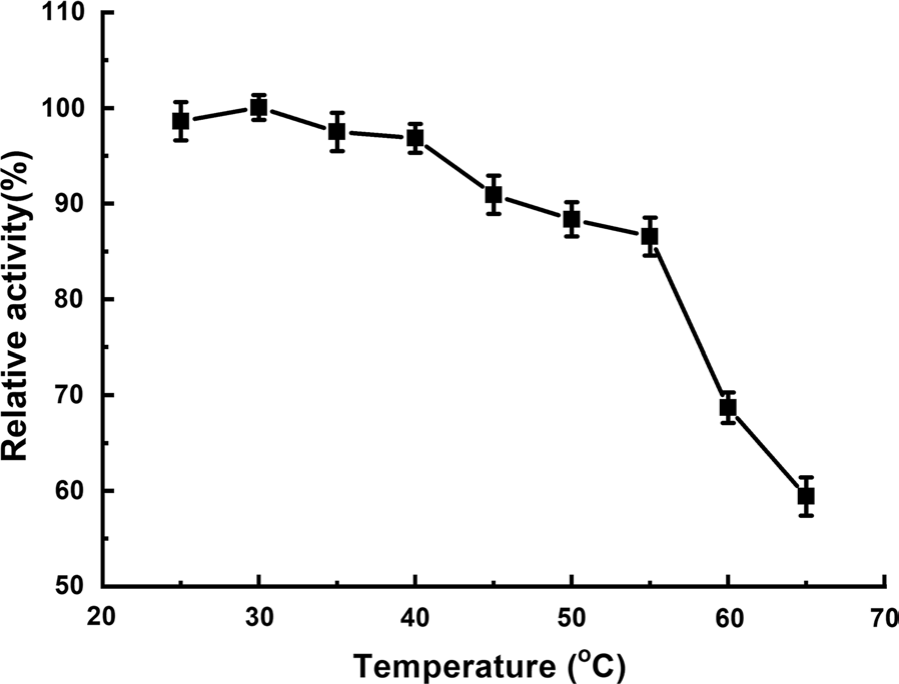
Effect of temperature on activity of esterase Tan410

**Fig. 6:**
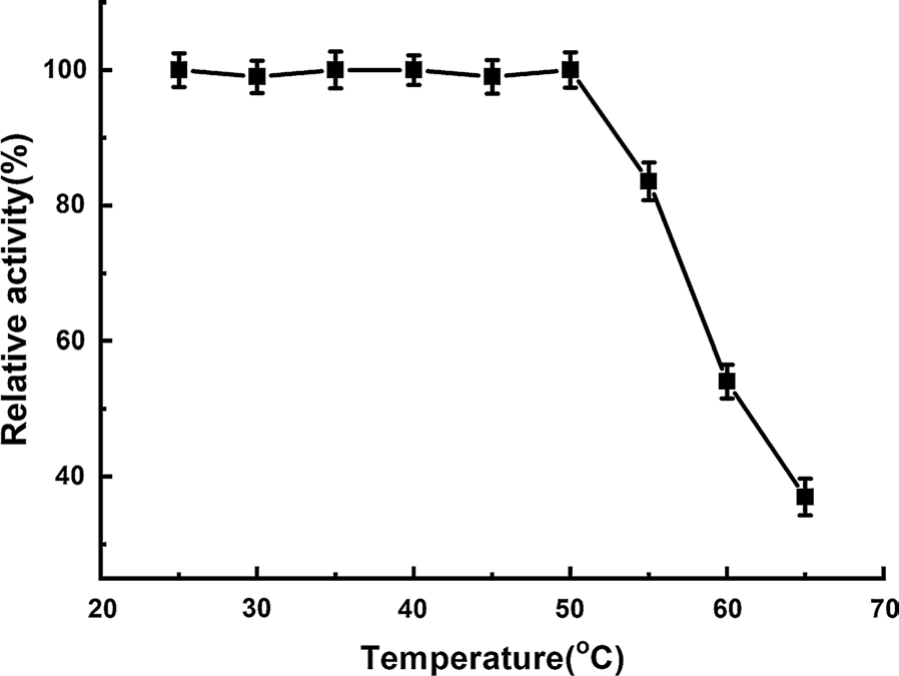
Effect of temperature on the stability of esterase Tan410

An ester library was used for testing substrate range of Tan410. And the highest hydrolytic activity was observed on vinyl butyrate, followed by ethyl hexanoate, phenyl acetate, isopropenyl acetate, ethyl octanoate and vinyl acetate. Tan410 also hydrolyzed other ester substrates, although showed less efficiency (such as ethyl butyrate, glyceryl triacetate, ethyl bromoacetate, methyl bromoacetate, glyceryl tributyrate, vinyl pivalate, isobutyl Acetate, ethyl acetate, propyl acetate and vinyl decanoate). However, Tan410 was unable to hydrolyze ethyl decanoate, ethyl oleate and methyl glycolate (Fig. 1). Esterase activity on purified Tan410 was confirmed using *p*-nitrophenyl esters possessing different acyl chain lengths from C_2_ to C_10_. Tan410 was active on all substrates assayed, exhibiting a clear preference for *p*-nitrophenyl acetate which was selected as substrate for determining biochemical properties of Tan410 (Fig. 2). The optimum activity of esterase Tan410 was measured over a pH range of 5.5–8.0 and temperature range of 25–65 °C. As shown in Fig. 3, esterase Tan410 showed its highest activity at pH 7.0, and more than 95% of initial activity remained after 1 h pre-incubation in pH range of 4.5–10.0 (Fig. 4). The optimum temperature of esterase Tan410 was found to be 30°C and the enzyme still possessed more than 90% residual activity not higher than 55 °C (Fig. 5). Thermostability of the enzyme was measured after pre-incubation at a temperature range of 35–65 °C. As shown in Fig. 6, it exhibited high thermostability and no activity was lost after heat treatment of temperature not higher than 50 °C. However, when the temperature increased, the enzyme activity decreased; it remained about 83%, 54% and 37% of initial activity after pre-incubation at 55 °C, 60 °C and 65 °C, respectively.

Effect of various chemicals on Tan410 activity was examined by pre-incubating the enzyme with chemicals in 50 mM phosphate buffer (pH 7.0) for 1 h (at 4 °C) and then measuring its residual activity using *p*-nitrophenyl acetate as substrate. Results (Table 1) showed that all test metal ions (Mg^2+^, Mn^2+^, Cu^2+^, Co^2+^, Zn^2+^, K^+^, Fe^2+^, Ni^2+^, Ca^2+^, Ag^+^ and Pb^2+^) demonstrated inhibition on the enzyme activity. Ag^+^ displayed more serious inhibition, remained only 4.5% of total activity. No activity was detected when SDS was added to the test solution. *N*,*N*- dimethyl formamide and ethanol slightly increased esterase Tan410 activity and methanol showed no affection on the enzyme activity. Esterase activity was slightly affected by ethyl acetate, isopropyl alcohol and acetone while highly inhibited by isoamyl alcohol.

**Table 1:** Effects of different chemicals on esterase Tan410 activity

Chemicals (5 mM)	Relative activity (%)
Control	100
Mg^2+^	83.2
Mn^2+^	67.6
Cu^2+^	79.5
Co^2+^	74.0
Zn^2+^	74.5
K^+^	87.4
Fe^2+^	56.1
Ni^2+^	64.7
Ca^2+^	80.3
Ag^+^	4.5
Pb^2+^	78.9
CTAB	11.5
SDS	0
PMSF	84.7
Organic solvent (10%)	
* N*,*N*-dimethyl formamide	105.6
Ethyl acetate	94.7
Isopropyl alcohol	98.5
Isobutyl alcohol	89.0
Isoamyl alcohol	37.4
Acetone	95.7
Methanol	100.1
Ethanol	105.1

### Analysis of Tan410 on different substrates

**Fig. 7:**
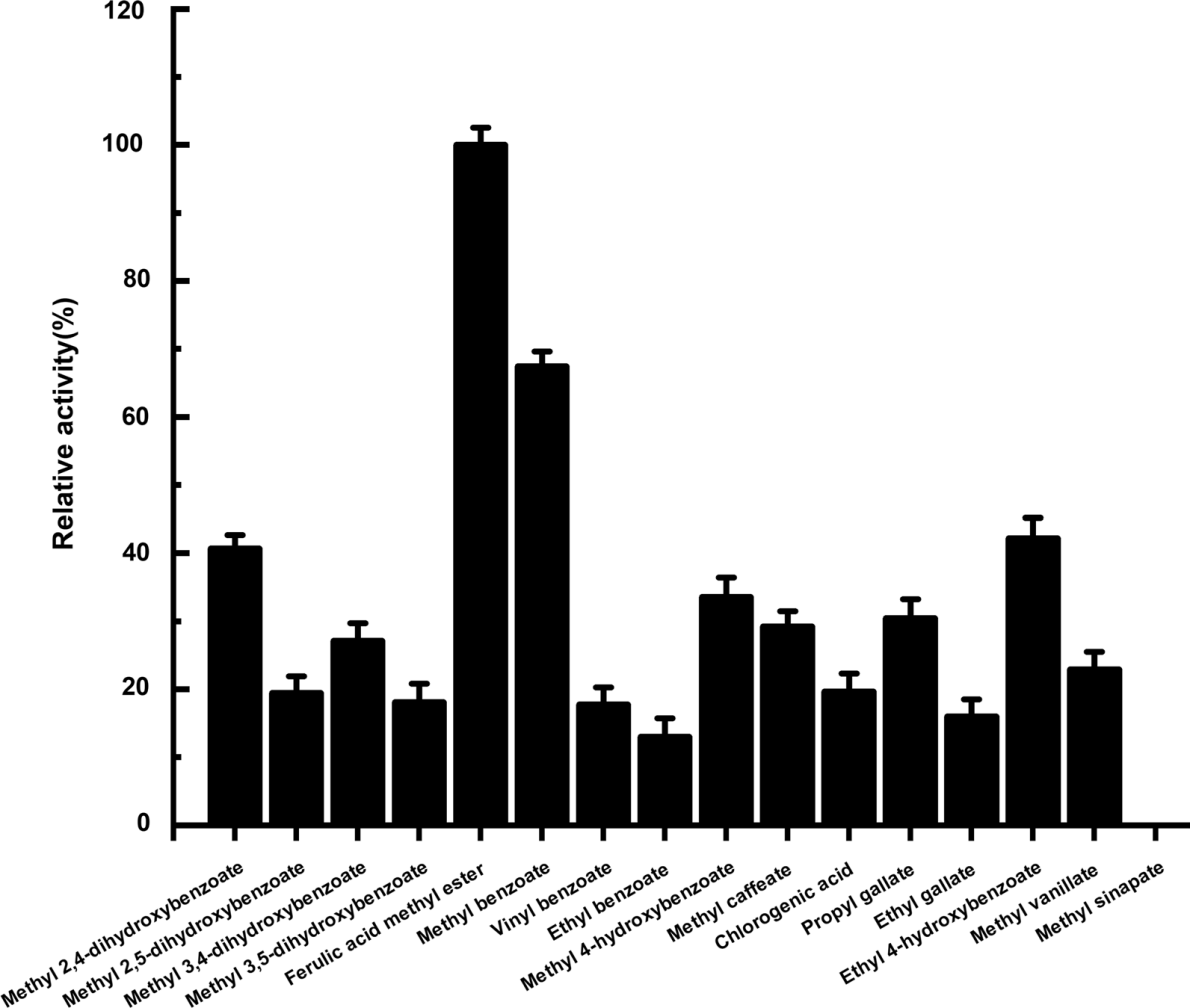
HPLC analysis of different substrates hydrolyzed by esterase Tan410

A comprehensive set of substrates was examined for hydrolysis by esterase Tan410. As shown in Fig. 7, the determined specific activity for methyl ferulate was set to 100% and used for the calculation of relative activities for all other substrates. All tested substrates (ethyl gallate, propyl gallate, methyl benzoate, ethyl benzoate, vinyl benzoate, methyl 4-hydroxybenzoate, ethyl 4-hydroxybenzoate, methyl vanillate, methyl 2,4-dihydroxybenzoate, methyl 2,5-dihydroxybenzoate, methyl 3,4-dihydroxybenzoate, methyl 3,5-dihydroxybenzoate, methyl ferulate, methyl caffeate, methyl *p*-coumarate, methyl sinapinate and chlorogenic acid) could be hydrolyzed by the enzyme. Methyl ferulate was found to be hydrolyzed most effectively, while methyl sinapate could not be hydrolyzed by Tan410.

## Discussion

Phenolic compounds, ubiquitous in plants were essential part of human diet, as they accounted for all most one-third of dietary phenols (Haminiuk et al. 2012; Esteban-Torres et al. 2015), which could be found in apple, artichoke, eggplant, grape, pear, potato and grape. They were also found in herbs such as eucommia, wormwood and honeysuckle (Santana-Gálvez et al. 2017). Phenolic compound had been reported to have anti-oxidant, anti-inflammatory, anticarcinogenic and antidiabetic activities. Therefore, enzymes which were able to release these compounds were not only essential for the degradation of plant but also of interest for the potential food and medical applications. In this study, a metagenomic library was constructed for screening esterase genes and a novel gene encoding multi-functional esterase was isolated. The gene (*Tan410*) encoding esterase was cloned and expressed in *E.coli* BL21(DE3) and the recombinant protein was purified for studying on enzymatic properties.

Substrates in a library were used for determining the substrate profile of Tan410. Based on activity observed, it could be concluded that Tan410 showed a wide substrate range. Furthermore, it was interesting to find that Tan410 could hydrolyze methyl ferulate, methyl *p*-coumarate and methyl caffeate, but not methyl sinapate, indicating that Tan410 showed feruloyl esterase activity and concluding that Tan410 was a type B feruloyl esterase. In addition, it was found that Tan410 could also hydrolyzed chlorogenic acid and rosmarinic acid, which was in accordance with feruloyl esterase from *Lactobacillus johnsonii* (Lai et al. 2009). A few fungus feruloyl esterases, such as feruloyl esterase from *Aspergillus niger* (Levasseur et al. 2004), AoFaeB and AoFaeC from *A. oryzae* (Koseki et al. 2009), TsFaeC from *Talaromyces stipitatus* (Vafiadi et al. 2006), and Fae1A from *Anaeromyces mucronatus* (Qi et al. 2011) had been reported to show activity toward chlorogenic acid. However, their activities against rosmarinic acid were not analyzed. Furthermore, Tan410 also exhibited activity against model hydroxybenzoic esters hydrolyzed by tannase (tannin acyl hydrolase), gallate and protocatechuate esters. Hydrolytic activity on esters from hydroxybenzoic acids was not a common activity on feruloyl esterases. To our knowledge, an enzyme Est_1092 possessing feruloyl esterase and tannase activity had been reported from from *L. plantarum* (Esteban-Torres et al. 2015). However, Est_1092 could not hydrolyzed chlorogenic acid and rosmarinic acid, while Tan410 was able to hydrolyze chlorogenic acid and rosmarinic acid.

In early reports, it was found that tannase was limited to hydrolyze esters derived from gallic and protocatechuic acids. Iibuchi et al. had studied about the intermediate hydrolysates of tannic acid, substrate specificity, and inhibition of tannase activity by substrate analogues. They found that only ester compounds of gallic acid could be hydrolyzed by tannase, except 3,4-dihydroxy methylbenzoate (methyl-protocatecuate) (Sadaaki et al. 1972). Ren et al. studied three-dimensional structure of a tannase from *L. piantarum*, and also studied catalytic and substrate binding sites. They found a catalytic triad (composed of Ser 163, His451, and Asp 419) in the structure and their mutagenesis studies revealed that residues in the catalytic triad and those interacting with three hydroxyl groups of the gallic acid were indispensable for the activity of tannase (Ren et al. 2013). The binding between hydroxyl groups of substrates and catalytic residues could be disrupted by mutation of catalytic triad residues, which caused the termination of catalytic reactions (Ren et al. 2013). Finally, they deduced that at least two of the three hydroxyl groups were needed to form a stable complex structure between the enzyme and a substrate (Ren et al. 2013). It seemed that tannase could not hydrolysis hydroxybenzoic acid without hydroxyl groups and hydroxybenzoic acid with substituents other than –H or –OH at position 2 (Esteban-Torres et al. 2015). However, in this study, it was interesting to find that Tan410 was able to hydrolyze benzoic esters (methyl benzoate, ethyl benzoate and vinyl benzoate) without hydroxyl group on the benzene group. Furthermore, it was also found that Tan410 could hydrolyze hydroxybenzoic esters (methyl 4-hydroxybenzoate and ethyl 4-hydroxybenzoate), dihydroxybenzoic esters (methyl 2,4-dihydroxybenzoate, methyl 2,5-dihydroxybenzoate, methyl 3,4-dihydroxybenzoate and methyl 3,5-dihydroxybenzoate) and vanillic ester (methyl vanillate). Therefore, Tan410 was not only a feruloyl esterase and tannase because of its hydrolyzation of esters from hydroxycinnamic and hydroxybenzoic acids, but also was an esterase active on a broad range of esters from phenolic acids, which was similar to that of Est_1092 esterase from *L. plantarum* (Esteban-Torres et al. 2015).

**Fig. 8:**
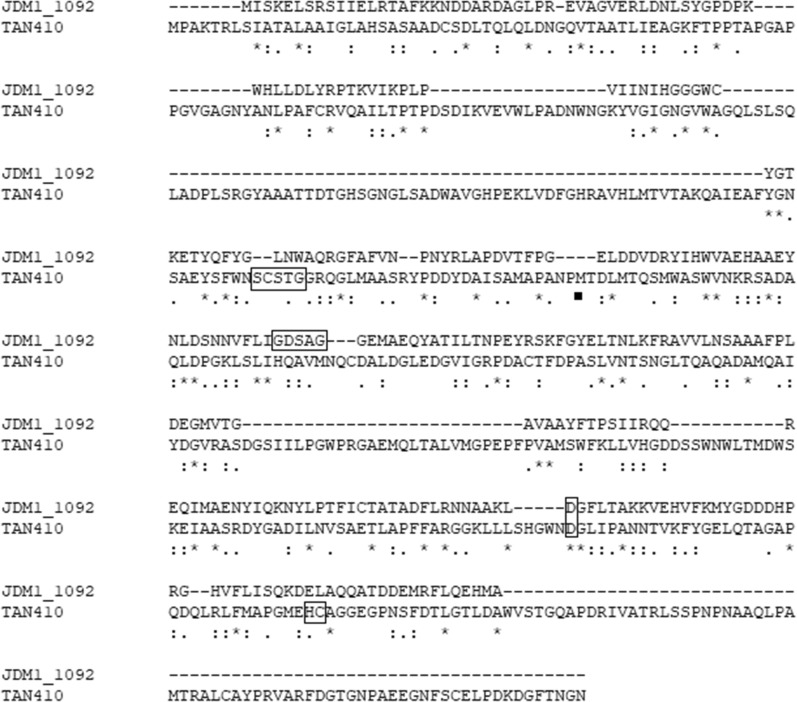
Sequence alignment of esterase Tan410 with Est_1092

As Tan410 was similar to Est_1092 in their enzymatic properties, the amino acid sequence of Tan410 and Est_1092 was compared to see whether they showed higher similarity. Unfortunately, their amino acid sequence showed lower similarity (36.36%). Amino acid sequence analysis revealed, that Est_1092 exhibited the conserved motif Gly-X-Ser-X-Gly typical of serine hydrolases which was converted to Ser-X-Ser-X-Gly in Tan410. Furthermore, structural motif CS-D-HC was found in Tan410 amino acid sequence, while it was not existed in Est_1092 amino acid sequence (Fig. 8). Maybe these features made Tan410 showed active on a broader range of esters from phenolic acids than that of Est_1092.

In conclusion, a gene encoding a multi-functional esterase Tan410 was isolated from a metagenomic library. Tan410 showed highest activity toward *p*-nitrophenyl acetate, indicating it was a typical carboxylesterase rather than a lipase. Beside *p*-nitrophenyl esters, substrate profile revealed that the enzyme could also hydrolyze hydroxycinnamic and hydroxybenzoic esters, suggesting Tan410 was an enzyme active on a broad range of phenolic esters, which made it a valuable candidate for biological applications.

## Supplementary Information

**Additional file 1: Figure S1.** Sequence alignment of esterase Tan410 with homologous sequences.

**Additional file 2: Figure S2.** SDS-PAGE analysis of recombinant Tan410. M, marker proteins, lane 1, purified Tan410.

## Data Availability

The datasets used and/or analyzed during the current study are available from the corresponding author on reasonable request.
