# Supplementary figures and images for: A novel esterase from a soil metagenomic library displaying a broad substrate range
Source: AMB Express. 2021 Mar 5;11:38. doi: 10.1186/s13568-021-01198-5 (PMC7936011; doi:10.1186/s13568-021-01198-5)

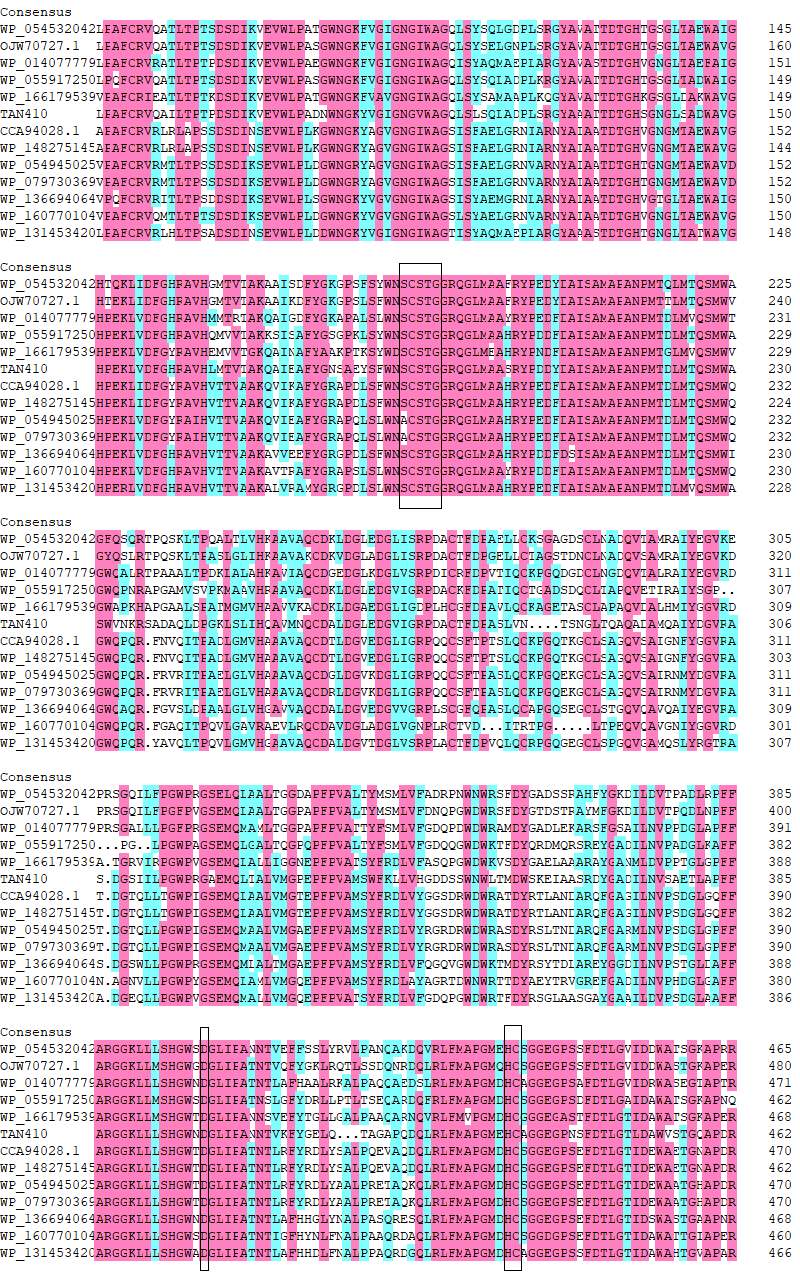

Supplement: Supplementary file 1 — Additional file 1: Figure S1. Sequence alignment of esterase Tan410 with homologous sequences. [file 13568_2021_1198_MOESM1_ESM.tif]

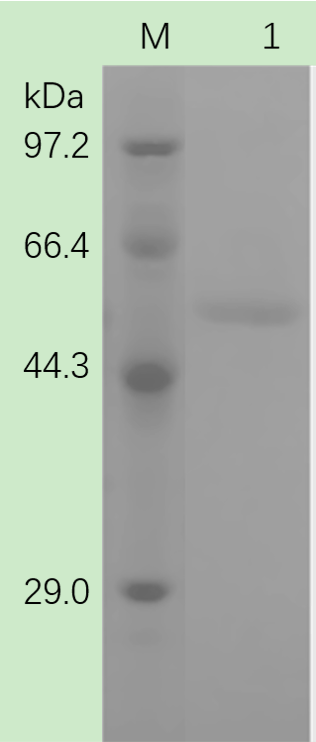

Supplement: Supplementary file 2 — Additional file 2: Figure S2. SDS-PAGE analysis of recombinant Tan410. M, marker proteins, lane 1, purified Tan410. [file 13568_2021_1198_MOESM2_ESM.tif]
